# Context-Dependent Neural Activation: Internally and Externally Guided Rhythmic Lower Limb Movement in Individuals With and Without Neurodegenerative Disease

**DOI:** 10.3389/fneur.2015.00251

**Published:** 2015-12-02

**Authors:** Madeleine E. Hackney, Ho Lim Lee, Jessica Battisto, Bruce Crosson, Keith M. McGregor

**Affiliations:** ^1^Atlanta VA Center for Visual and Neurocognitive Rehabilitation, Decatur, GA, USA; ^2^Division of General Medicine and Geriatrics, Department of Medicine, Emory School of Medicine, Atlanta, GA, USA; ^3^Emory College of Arts and Sciences, Emory University, Atlanta, GA, USA; ^4^Department of Neurology, Emory School of Medicine, Atlanta, GA, USA

**Keywords:** lower limb, motor control, neuroimaging, rhythm, externally cued, internally guided, Parkinson’s disease

## Abstract

Parkinson’s disease is a neurodegenerative disorder that has received considerable attention in allopathic medicine over the past decades. However, it is clear that, to date, pharmacological and surgical interventions do not fully address symptoms of PD and patients’ quality of life. As both an alternative therapy and as an adjuvant to conventional approaches, several types of rhythmic movement (e.g., movement strategies, dance, tandem biking, and Tai Chi) have shown improvements to motor symptoms, lower limb control, and postural stability in people with PD (1–6). However, while these programs are increasing in number, still little is known about the neural mechanisms underlying motor improvements attained with such interventions. Studying limb motor control under task-specific contexts can help determine the mechanisms of rehabilitation effectiveness. Both internally guided (IG) and externally guided (EG) movement strategies have evidence to support their use in rehabilitative programs. However, there appears to be a degree of differentiation in the neural substrates involved in IG vs. EG designs. Because of the potential task-specific benefits of rhythmic training within a rehabilitative context, this report will consider the use of IG and EG movement strategies, and observations produced by functional magnetic resonance imaging and other imaging techniques. This review will present findings from lower limb imaging studies, under IG and EG conditions for populations with and without movement disorders. We will discuss how these studies might inform movement disorders rehabilitation (in the form of rhythmic, music-based movement training) and highlight research gaps. We believe better understanding of lower limb neural activity with respect to PD impairment during rhythmic IG and EG movement will facilitate the development of novel and effective therapeutic approaches to mobility limitations and postural instability.

## Rehabilitation in Parkinson’s Disease

Pharmacology and surgery do not fully address the motor, cognitive, and psychosocial needs of those with Parkinson’s disease (PD), a neurodegenerative disorder that is related to dopamine depletion in the substantia nigra pars compacta, which in turn hinders processing in the basal ganglia (7). Several mobility programs are effective (e.g., mobility training, dance, tandem biking, and Tai Chi) for improving motor symptoms, lower limb control, and postural stability in people with PD (2–6). These programs use a mixture of internally guided (IG) and externally guided (EG) movement strategies, both of which have evidence to support their use in rehabilitative scenarios. However, little is known about rhythmic lower limb movement relating to locus of cue (EG vs. IG). Many rehabilitative programs preferentially select one locus over another, which may or may not be optimal for long-term improvement of mobility. Better mechanistic understanding of beneficial exercise effects on neural circuitry garnered under specific contexts could improve the design of motor rehabilitation interventions for particular symptoms (e.g., freezing and bradykinesia) and the various disease stages of PD.

The goal of this review is to provide rehabilitative specialists and researchers with the state of the rehabilitation science regarding the potential neural underpinnings of IG and EG movements. Respective of this, IG movement neural dynamics will be contrasted with those of EG movements. As implemented, the dichotomy provided in this review respective of movement locus of cue is didactically necessary but practically difficult to realize from a rehabilitative perspective. Ecologically speaking, human movement is rarely, if ever, purely IG or EG, whether in the case of daily activities or in rehabilitative settings. That said, determining the beneficial and most effective qualities and outcomes of IG and EG motor training could inform rehabilitation particularly for largely intractable conditions like PD.

## Internally Guided Movement in Rehabilitation

Proper completion of IG movements relies on efficient function of subcortical loops involving the basal ganglia (8, 9). Due to dysfunction of the striato-thalamo-cortical (STC) circuit [also referred herein as the cortico-basal-ganglia-thalamic (CBGT)], people with PD have particular difficulty with IG tasks (10–12). However, this impairment can be remediated by motor rehabilitation that uses skills in which participants engage cognitively in planning and selecting movements (5). Specifically for individuals with PD, having complex movements broken down into simpler elements may facilitate motor performance. Employing a “movement strategy” that demands increased focus on movement plans and mentally rehearsing and/or preparing for self-initiated movement may be helpful. For example, focusing on critical movement aspects (e.g., longer steps, quicker movements) helps individuals with PD to achieve nearly normal speed and amplitude (13). Thus, IG training may be helpful for individuals with PD.

## Externally Guided Movement in Rehabilitation

Abundant evidence also demonstrates benefits of rehabilitative exercise that exploits external cueing, which likely specifically targets neural systems that support balance (4, 14). EG strategies have improved movement initiation (15, 16). Other research has shown that people with PD have faster reaction times when externally cued compared to self-initiated (IG) movement (17). Synchronizing movement to rhythmic beats provided externally may enhance movement speed (18). There is also a well-known facilitating effect of cues for alleviating freezing of gait (FOG) (19). Furthermore, gait training with regular external rhythmic auditory cues has improved gait velocity, stride length, step cadence, timing of EMG patterns, and mobility in persons with PD (20–22). Evidence has begun to accumulate that suggests external cues access alternate neural pathways that remain intact in the individuals with PD, including the cerebellar-thalamo-cortical (CTC) network.

## Characterization of the Neural Circuitry Involved with Internally and Externally Guided Movement

This review covers available literature focused on imaging studies involving IG and EG upper and lower limb movement paradigms within the contexts of cortical and subcortical neural function. Our goal is to summarize findings from the available lower limb literature to inform future research with goals of characterizing neural areas involved in motor rehabilitation of PD. Numerous neural systems likely produce IG and EG movement and could be modulated by rehabilitative training. However, the current work is not intended to provide an encyclopedic reference to neural network function in motor systems, although we provide a reference in Table 1 that catalogs a number of studies that involve IG and EG paradigms. Rather we will focus on the neural routes that likely assume multiple subcomponents to be explicated in future rehabilitation research. These routes are (a) cortically modulated (mainly in the frontal and parietal lobes), (b) subcortically modulated including the basal ganglia and thalamus, and (c) the cerebellum.

**Table 1:** **A summary of the relevant imaging studies in the context of IG vs. EG movement in healthy controls and individuals with Parkinson’s disease**.

Reference	Population and *N*: (young <40 years; older >50 years)	Internally guided or externally guided	Upper or lower limb movement	Task	Finding
(23)	12 Healthy young	EG	Lower limb	Imagery and execution of ankle dorsiflexion	EG movement execution and motor imagery shared a common network, including the premotor, parietal and cingulate cortices, the striatum, and the cerebellum
(24)	4 Hemiparetic vs. 12 healthy subjects (25–70 years)	EG	Lower limb	Ankle dorsiflexion	EG-guided hemiparetic
(25)	20 PD patients; 10 healthy (non-age matched)	EG	Lower limb	Ankle dorsiflexion	PD-off: precentral gyrus, supplementary area, parietal opercular cortex, and ipsilateral cerebellum activated; PD-on: similar activation pattern as off, with additional activation of insular cortex; healthy-off: contralateral precentral gyrus, central opercular, cortex, and ipsilateral cerebellum; healthy-on: activations in precentral gyrus, central and parietal opercular cortex, cerebellum, and posterior cingulate cortex – no sig. increased activation in on vs. off for controls
(26)	8 Healthy (25–57 years)	EG	Lower limb	Ankle dorsiflexion vs. plantarflexion	EG dorsiflextion activated from medial M1S1 to SMA
(27)	16 Healthy young	EG	Lower limb	Ankle dorsiflexion vs. plantarflexion	Both right and left ankle active movements activated SMA, contralateral M1, and primary somatosensory cortex (SI)
(28)	13 PD, 13 age-matched healthy	IG	Lower limb	Gait imagery	During imagined movement, right dorsal premotor area (PMd), precentral, right inferior parietal lobule, and bilateral precuneus were more activated in PD compared to age-matched controls
(29)	18 Healthy young	IG and EG	Lower limb	Ankle dorsiflexion with and without visual cue	IG ankle movements has distinct network comprising the posterior parietal cortex and lateral cerebellar hemispheres
(30)	16 Healthy young	EG	Upper and lower limbs	Wrist and ankle flexion	Lower extremity EG more bilaterally active than upper extremity EG
(31)	24 Healthy young	EG	Upper and lower limbs	Foot and finger movement	Relative overlap of cortical recruitment in M1 and SMA for lower extremity and upper extremity movements
(32)	23 Healthy young	IG	Upper and lower limbs	Adduction and abduction of finger vs. adduction and abduction of foot	Cerebellum: overlap of activations for foot and finger movement
(33)	17 Healthy young, 21 healthy older	IG	Upper and lower limbs	Hand and foot flexion	Older adults recruited a more elaborate network of motor and non-motor regions younger adults
(34)	13 Healthy young	IG and EG	Upper and lower limbs	Finger vs. toe flexion	Finger and toe movements showed differential cerebellar recruitment; more bilateral during complex tasks
(35)	11 Healthy young	EG	Upper limb	Hand force production	Caudate nucleus is involved in planning motor force, but not force execution in EG tasks
(36)	10 Healthy young	EG	Upper limb	Finger button press and motor imagery	Cognitive and motor processes activate segregated areas of the cerebellum
(37)	9 Healthy young	EG	Upper limb	Finger tapping	EG finger tapping recruited cerebellum: right lobules IV–V and right lobules VIIIA and VIIIB
(38)	7 Healthy young	EG	Upper limb	Finger button press	Finger specific BOLD patterns showed overlapping sensory and motor representations in cerebellum
(39)	11 Healthy young	EG	Upper limb	Hand force production	Only the caudate nucleus increased activation when the subjects mapped force
(40)	10 PD; 10 age-matched healthy	EG	Upper limb	Hand force modulation	Off medication PD subjects have novel area recruitments of the bilateral cerebellum and primary motor cortex as compared to healthy adults
(41)	10 Healthy young	IG and EG	Upper limb	Drawing vs. tracing with hand	Results indicated that compared to tracing (EG), drawing (IG) generated greater activation in the right cerebellar crus I, bilateral pre-SMA, right dorsal premotor cortex, and right frontal eye field
(42)	32 PD patients; 16 w/FOG, 16 w/o FOG	IG	Upper limb	Finger flexion	An upper extremity task reveals differential striatocortical involvement for successful movements with PD patients with and without FOG
(43)	14 Healthy young	IG and EG	Upper limb	Finger button press	Our results show that the putamen is particularly involved in the execution of non-routine movements, especially if those are self-initiated
(44)	6 PD and 6 healthy	IG and EG	Upper limb	Finger flexion during positron emission tomography	Compared to PD patients, healthy adults showed greater activation of SMA and anterior cingulate, left putamen, left insular cortex, right DLPFC, and right parietal area 40
(45)	12 Healthy young	IG and EG	Upper limb	Phasic movements of hand vs. foot	During EG, the hMT/V5+, the superior parietal cortex, the premotor cortex, the thalamus, and cerebellar lobule VI showed higher activation. During IG: the basal ganglia, the SMA, cingulate motor cortex, the inferior parietal, frontal operculum, and cerebellar lobule IV–V/dentate nucleus showed increased activity
(46)	5 PD and 5 age-matched healthy	IG and EG	Upper limb	Finger tapping	PD patients showed increased recruitment of ipsilateral CTC circuit during EG task than healthy older adults
(47)	10 PD; 10 age-matched healthy	IG and EG	Upper limb	Finger tapping	Differential activation between PD and older adults during synchronous movements
(48)	10 PD; 13 age-matched healthy	IG and EG	Upper limb	Finger tapping	Eg movement showed decreased activation in the M1S1, cerebellum, and medial premotor system in PD subjects compared to healthy controls
(49)	10 Healthy young	IG and EG	Upper limb	Hand force production	IG: anterior basal ganglia more heavily recruited; EG: cerebellum more heavily recruited
(50)	35 Healthy (21–67 years)	IG and EG	Upper limb	Finger flexion	Differential recruitment of CBGT and CTC circuits respective of mapping of “what” or “when” during IG vs. EG tasks

**Figure 1:**
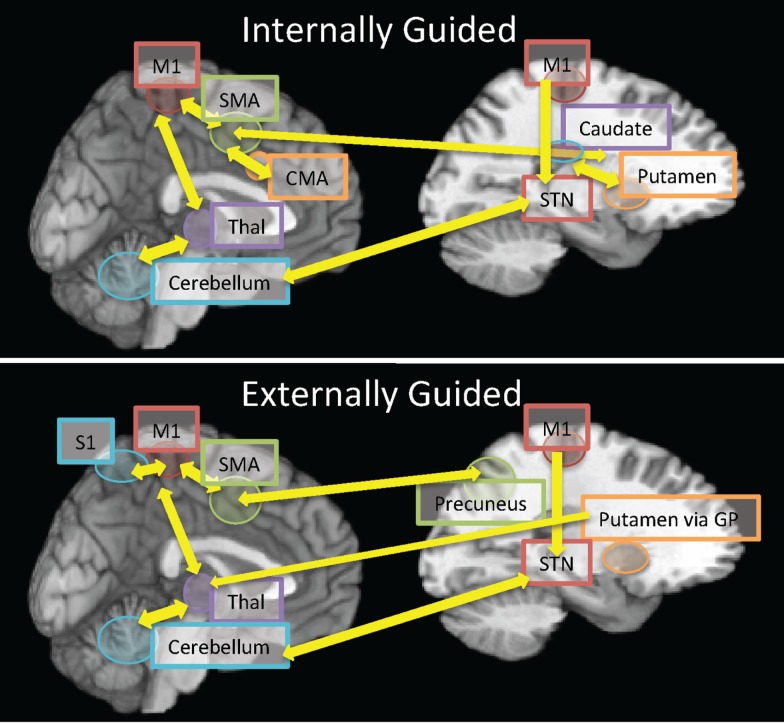
**A representative synopsis of the connectivity reported in the current review comparing neural connectivity in internally guided vs. externally guided movements**. The top panel represents connectivity within internally guided movements whose initiation from cortical regions (SMA, M1, and CMA) is mediated by the striatum (caudate for movement selection; putamen for execution) and thalamo-cerebellar bidirectional processing for movement execution. By contrast, the bottom panel represents connectivity during externally guided movements, which originate from sensorimotor integration (M1S1, SMA, and precuneus) due to the external cue for initiation. The progression of motor execution engages the lentiform nuclei (putamen, GP), which then influence cortico-thalamo- cerebellar processing during task execution. Abbreviations: SMA, supplementary motor area; M1, primary motor cortex; S1, primary somatosensory cortex; STN, subthalamic nucleus; CMA, cingulate motor area; GP, globus pallidus; Thal, thalamus.

In the neurotypical model, the investigation of IG and EG movements of the upper extremity has received considerable attention in neuroimaging (41, 46, 49, 51, 52). These studies have suggested distinct cortico-cerebellar, cortico-cortico, and cortico-subcortical neural pathways for IG vs. EG in a variety of contexts. The following sections will address differences between neural activity from both a region of interest (ROI) and neural network perspective. It needs to be noted that a vast majority of motor-related literature with such a focus has been done in upper extremity movements. Given the relatively young science of neuroimaging, this is somewhat understandable as there are considerable technical difficulties in controlling motion being translated from the legs to the head during movement. As such, studies investigating neural correlates of movements of the lower extremity in humans in the context of IG vs. EG control are rare. We will attempt to address the differences between upper extremity movements and lower extremity movements as available literature can inform. However, the scarcity of data involving lower extremity movements does not afford us a complete understanding of the potential variability explained by contrasting upper vs. lower limb movements. While Keele et al. (53) demonstrated that finger tapping, forefoot tapping, and heel tapping are highly correlated in light of timing mechanisms (53), differences between hand and foot movements in functional brain anatomy have been reported in imaging studies (54, 55). An important example of these differences was recently reported by Volz et al. (30) who used functional magnetic resonance imaging (fMRI) to assess cortical network function in younger adults performing unimanual hand or foot tapping. The authors reported significant differences in premotor connectivity with hand movements having a much stronger cortical representation in the motor planning areas. Additionally, upper extremity performance appears to be highly lateralized as compared to lower limb movements. With respect to disease models, the examination of motor control in conditions like PD has largely focused on upper limb control. Respective of this, where applicable and available, we will attempt to differentiate reports involving lower extremity from upper extremity research (Figure 1).

## Internally Guided Neural Circuitry – Cortical, Subcortical, and PD

### Cortical Activity

Cortical initiation of IG movements has received a fair amount of study with neuroimaging over the past 20 years. Prior to this, based on primate models, the role of the supplementary motor cortex (SMA) was considered crucial to the initiation of IG movements. Single unit recordings of neurons in macaque SMA showed greater spiking during IG movements rather than those prompted by external cues, which were thought to involve premotor areas (rostral to the anterior commissure in BA6) (56, 57). However, as our understanding of neural circuits improved with neural pathway tracings in primates, anatomical differentiation of these regions informed the functional distinction of premotor cortex, pre-SMA, and SMA (58, 59). When neuroimaging techniques were applied to probe the anatomical substrates involving IG movements, a much different picture with respect to cortical involvement emerged. Multiple labs used fMRI during upper extremity tasks to identify cortical activity during IG movement paradigms and found that the SMA (medial BA6, posterior to the anterior commissure) was not significantly driving the execution of movements in IG conditions (49, 60). Instead, there was a strong influence of a complex cortical and subcortical initiation and gating network that has since been largely confirmed by network-based modeling analysis (39, 50). Moreover, recent work has identified functionally distinct networks respective of the timing of the IG movements with alteration of cortico-basal ganglia involvement during initiation and then execution of the movement. During movement initialization (i.e., the process of identification/selection of motor responses), the anterior cingulate motor area (which may be considered an extension of pre-SMA) and pre-SMA appear to be critically involved as their damage results in improper suppression of selected motor responses (50, 61). However, the transition into direct engagement of skeletal muscle recruits additional cortical resources including lateral BA6, inferior parietal regions, and lateral inferior frontal areas (Broca’s area – left BA44) (62). This lateral frontal recruitment could be interpreted as selective movement organization, as Broca’s area is crucial for proper syntactic discourse (63, 64). For example, in the English language, syntax is heavily dependent on word order, and even more so in German. Broca’s area is critical in identifying the sequential order of word placement (63). In an analogous motor role, activation in Broca’s area may indicate order selection for organized movement (65).

### Subcortical

The cortical components of IG are strongly gated and in some cases are dependent on reciprocal input from subcortical structures, primarily the striatum. The caudolenticular gray bridges, topographically interposed between the caudate and the putamen, function as the primary efferent center from pre-SMA to the basal ganglia (66). As such, the striatum plays a critical role in modulating movement, and its activity is largely dependent on movement state. A functional dichotomy exists between caudate and putamen activity during IG movements. The caudate is linked to motor learning and sensory processing of proprioceptive input in this context (52, 67). Vaillancourt et al. (39) reported on IG force production at different levels during fMRI (39). Interestingly, the caudate was selectively activated only during the process of identifying and selecting the proper force to produce. Later, the group exquisitely showed that the caudate was selectively engaged in the processing of force for production but did not activate during force production (35). As such, the caudate is likely directly involved in the mapping of higher cortical motor process [input to the gray bridges is through premotor, SMA and M1 connectivity in IG movements (68)]. However, the caudate does not appear to assist in timing of IG movement execution. Instead, this function appears to be fulfilled by the putamen.

Many imaging studies have probed the function of the putamen and its role in timing of IG movements. For years, this structure has been reported as active during execution of IG tasks using fMRI (9, 35, 39, 49, 67, 69). Recently, these reports have been informed by alternate modalities indicating the neural timing of activity in the putamen with respect to regulation of IG movement. Using local field potential (LFP) recordings in macaques, Bartolo et al. recently investigated tuning of spiking potentials respective of movement state, either EG or IG cued (70). While the putamen is involved in both types of activity, the waveforms of the spike potentials tuned differentially to the locus of cue. These waveforms were characterized by frequency consisting of beta (13–30 Hz) and gamma frequencies (30–70 Hz) and were compared during the execution of IG and EG tasks. IG movements were strongly aligned with the beta band of the LFPs. Alternately, during EG movements, LFP activity was characterized by gamma frequency oscillations. Importantly, when sampling across larger distances the beta frequency coherence was high indicating a possible functional coordination of the putamen during IG tasks. Conversely, the spatial coherence of gamma frequencies was very low, which could be interpreted to indicate local, single event processing (70, 71). The authors concluded that the gamma frequency activity potentially indicated local computations directly related to external stimulus processing. In contrast, the beta band preference in the putamen was the result of a larger scale entrainment of CBGT circuits as these were associated with IG movement timing. The authors provided additional evidence for this in a recent paper investigating LFP oscillations during different task types (reaction time vs. internally timed tapping) (72).

#### Cerebellum

Although we have thus far focused on the STC circuit in its role for IG movement, the cerebellum has been implicated in IG movement as well. While this may be surprising given the literature recognizing cerebellar involvement in explicitly EG tasks (41, 45, 47, 60, 73), IG tasks have been reported to recruit cerebellar regions. fMRI studies involving IG movements of the upper extremity have reported regional recruitment of cerebellum, including lobulus V (anterior cerebellum) and lobules VIIB and VIII (inferior cerebellum) (74). During arm pointing movements (using both left and right arms), activations were found ipsilateral to movement in lobule V and lobule VIIA (34). While studies employing lower extremity movement during IG tasks are scarce, a study from Schlerf et al. (34), in which they attempted to characterize cerebellar activity during IG upper and lower extremity movements, is worth noting. The group investigated differential cerebellar involvement when modulating task difficulty of both the upper and lower extremities (fingertip and toe, respectively). Toe tapping activated more anterior areas of the cerebellum compared to finger movements regardless of movement difficulty. Interestingly, the group also found greater bilateral cerebellar activity during more difficult lower extremity movements compared to analogously difficult conditions during finger tapping. Other groups have further investigated the possibly differential cerebellar representations of lower limb movement as contrasted to upper extremity movement. A recent study by Kuper et al. (32) aimed to identify overlapping or distinct cerebellar activity when contrasting finger and foot tapping at the joints’ maximum movement range (32). Interestingly, while overlapping cerebellar finger and foot activity were present [cf., Ref. (34)], Kuper et al. found activity appeared to follow a somatotopy with foot activation occurring more rostrally compared to finger movement.

However, the attribution of cerebellar recruitment with respect to IG vs. EG locus of cue is incomplete without proper conceptualization of timing within a movement context. In a recent consensus paper (75), Richard Ivry denoted an important qualifier of cerebellar activity respective of movement timing, by noting that much work using imaging of the cerebellum has difficulty teasing apart externally cued movement and its transition to emergent timing. The difficulty is partially explained by the challenge that lies in differentiating absolute time vs. perceived time and the role that the cerebellum plays in optimizing the coordination of the two. Taken in this light, the role of cerebellar recruitment during IG vs. EG movement becomes somewhat mottled in the imaging literature. What may be deemed “internally guided” may be continuously informed by an emergent timing within the participant when this individual is trying to synchronize internal timing to his/her perception of absolute timing.

## Internally Guided Movement in Parkinson’s Disease

As PD is caused by the loss of endogenous dopamine, the disease profoundly affects basal ganglia function. The most immediate impact of dysfunction in IG movement in PD relates to impaired cortico-basal ganglia communication driving initiation of movement. One such behavioral manifestation of this disruption is the presence of bradykinesia in PD during IG movement (76). As stated above, cortical initiation of IG movements relies on basal ganglia modulation (particularly at the striatofrontal interconnect at the post-commisural putamen) and signal augmentation that is highly sensitive to disruption when filtered through dysfunctional lentiform connections (77). However, this physiology is complicated by the importance of sensory feedback required for proprioception and kinesthetic integration. These afferent inputs are directly modulated by thalamo-cortical relays involving the subthalamic nucleus, ventrolateral, and centromedian thalamic nuclei (68, 78, 79). As such, disruption of the sensory integration back to motor planning cortex further complicates successful movement initiation by disruption of physical state monitoring required for movement.

Deficient gating of sensory signals in the basal ganglia (80) may lead to abnormal processing of proprioceptive input in motor regions, such as the SMA (81). In a related study, Goble et al. (82) examined how brain activity resulting from stimulation of proprioceptors is related to performance in a proprioceptor-related task (82). Subjects lying in an fMRI scanner received vibrations on their foot, allowing for proprioceptive brain mapping via muscle spindle stimulation. Exposure to foot vibrations showed an association of the basal ganglia structures with structures involved in postural control. Movement studies show the basal ganglia play a significant role in motor learning. In a study by Jueptner and Weiller, positron emission tomography (PET) measurements of regional cerebral blood flow (rCBF) were used for studies of motor learning, visuomotor coordination, and sensory movement control (9). Furthermore, the basal ganglia have been shown to be involved in controlling ongoing movements, including feedback processing (83). Maschke et al. examined the role of the basal ganglia in kinesthesia, the conscious awareness of limb position, with a passive elbow movement task in participants with PD, participants with spinocerebellar ataxia (SCA), and age-matched healthy control participants. The PD participants showed significant kinesthetic deficit to control participants, whereas the SCA participants showed no kinesthetic deficit, allowing the conclusion that CBGT loops are important in kinesthesia (84).

Symptoms associated with PD provide some insight into the disease process on neural circuits and inform rehabilitation strategies of lower limb-related problems. FOG is a common and dangerous condition in individuals with PD whereby the person successfully initiates walking, but it is transiently unable to complete the gait cycle, frequently resulting in imbalance and increased fall risk. FOG is functionally distinct from bradykinesia and postural rigidity and is only conditionally affected by pharmacological treatment (85, 86). Although FOG is challenging to characterize as EG or IG, we will consider FOG a failure to continue a self-initiated movement, the primary manifestation of the condition. FOG very likely represents a disrupted sense of internal rhythmic timing (87). Peterson et al. (88) informed upon the brain regions that are involved in PD patients with and without FOG. In this study, PD patients imagined walking during fMRI acquisition. The results showed significantly lower activity in supplementary motor regions, globus pallidus, and cerebellum in individuals with PD who experience FOG compared to those who did not report FOG. Furthermore, the authors reported decreased activity in the mesencephalic regions associated with postural stability (88). These widespread activity differences between groups indicate that individual discrete neural circuits do not easily account for the motor dysfunction exhibited by FOG. Rather, cortical, subcortical, and cerebellar loops are all affected in FOG. This finding may explain the large variability of pharmacological and surgical outcomes relating to FOG in PD (85, 86, 89). Importantly, this study indicates rehabilitative strategies that are focused on IG movements need to be holistic in approach because no single sub-circuit or structure is preferentially involved in these movements.

## Externally Guided Neural Circuitry – Cortical, Cerebellar, and PD

Externally guided movements involve both overlapping and discrete brain regions for successful task completion. While studies vary widely in terms of the methodology employed, externally cued movement of differing types involves similar cortical, subcortical, and cerebellar substrates. We will explore these areas as they are associated with EG movements and discuss how PD may disrupt proper execution with implications for rehabilitation programs to follow.

### Cortical Involvement in EG Movement

Prior to modern neuroimaging, the initial cortical origin of IG movements was thought to be SMA (57), as cortical activity in EG was viewed in the context of the motor planning and, as such, focused on the relationship between premotor areas with primary motor cortex (90–92). This work in the non-human primate model still provides invaluable insight into cortical function, but it has been updated in the human model with functional neuroimaging. The following text details the differential recruitment of cortex in EG as contrasted to IG in light of modern imaging techniques.

In 2006, Elsinger and colleagues published an important paper differentiating IG and EG cortical dynamics during an upper extremity button press task in fMRI (60). Indeed, this group did show that lateral premotor areas were more active during EG response; however, this activity was likely part of a spatial reaction network associated with the monitoring of external stimuli. This network involves the right hemisphere parietal–premotor–frontal eye field regions (93). This monitoring network can be interpreted exquisitely due to the task-selective nature of the regions recruited. The frontal eye fields are critical in processing saccades and act as a visual motor integration respective of higher-level visual input. The spatial processing of object location is strongly associated with right parietal regions, which would necessarily inform premotor areas to prime selection and then execution of accurate motor response. It has been shown that increased task complexity corresponded to an increase in network coherence (60). Because the task was mapping of specific finger movements to external stimuli, the increased activation of this circuit as a result of increased complexity may be interpreted as multisensory integration of proprioceptive, visual, and kinesthetic information.

Cortical recruitment in EG tasks can also be described in the context of movement entrainment or sequence learning. Much attention has been paid to the role of EG in movement training to automaticity (94–97). Using PET, Jenkins et al. (95) was one of the first groups to use neuroimaging techniques to probe motor skill acquisition and the neural correlates that underlie the stages of motor learning (novel stimulus, movement entrainment, and automatic movement). Overall, the cortical representation pattern was characterized as moving from anterior frontal regions to more posterior areas after continued task practice. Specifically, the group denoted that novel motor learning was the only task condition to show activity in the prefrontal cortex. The entrainment phase of motor practice was associated with consistent activity within the SMAs [see also Ref. (98)], while at movement automaticity (maximum performance accuracy), the dominant cortical activity was in the parietal lobe (95). Work involving fMRI has also shown this anterior to posterior shift in cortical recruitment as tasks become well practiced (96) with an overall reduction of volume of cortical regions recruited after automaticity is achieved.

Importantly, the paradigms denoted thus far have only employed upper extremity movements. Much less is known about cortical execution of EG movements in contrast to IG respective of lower extremity movement. Bruce Dobkin’s laboratory has published much of the rehabilitation-focused imaging research on the lower extremity. In a series of studies involving the use of lower extremity EG movement, this team denoted reliable recruitment of left SMA, bilateral sensorimotor areas (M1S1), and right parietal lobules (24, 99). Other labs have also approached lower limb movement with considerations for rehabilitative outcome. For example, Trinastic et al. (26) attempted to differentiate cortical recruitment in motor cortex during EG plantarflexion as compared to dorsiflexion. Findings showed that although dorsiflexion recruited additional cortical areas as compared to plantarflexion, the tasks commonly recruited medial SMA (26). Kapreli et al. (100) approached lower limb movements relating to motor overlap of the somatosensory network shown in upper extremity movements. In this study, the group reported similar regions of activity between tasks; however, lower limb movements were much less lateralized and tended to recruit both hemispheres during motor activity (100). Laterality differences between upper and lower extremities have been reported during visual monitoring (101) and motor tracking tasks (102) comparing movements of the hand vs. the foot. This increased bilateral response [also reported by Trinastic et al.(26) during dorsiflexion as compared to plantarflexion] in lower extremity tasks as compared to those involving the upper extremity has likely been noted because locomotion requires bilateral coordination to maintain balance (26, 103). This differentiation also provides an opportunity for rehabilitation specialists to design interventions that take advantage of the greater cortical recruitment of lower limb movements.

### Subcortical Recruitment in EG

Although there exists regional differentiation of cortical recruitment respective of EG vs. IG movements, the neural system does not function as wholly discrete neural compartments as characterized thus far. Understanding the integration of subcortical and cerebellar structures in light of movement circuits is crucial to properly inform rehabilitation using IG and EG strategies in movement.

As stated earlier, sensory integration during EG task execution is a central component to neural activity in this modality. Without continuous state monitoring (postural, positional, visual, auditory, etc.), coordinating movement with external cues would be impossible. As such, the basal ganglia and thalamic sensory relay centers are critical components to successful EG task execution. To this end, in healthy individuals performing EG tasks, a complex interaction of thalamic (pulvinar, ventroanterior lateral, and ventroposterior lateral), cerebellar, and cortical regions form sensory circuits, which allow for selected task actions to coordinate with cues.

Taniwaki et al. (104) used fMRI to characterize discrete cortico-subcortical circuits associated with IG vs. EG movements in the upper extremity. The group employed a structural equation modeling (SEM) approach with *a priori* network structures identified to test for path strength between ROIs during EG compared to IG. The group identified discrete ROIs of active structures regardless of movement type before entering the regions into a confirmatory structural equation model. In reference to previous literature and task-based activation in the study (left hand movements), the group identified IG movements with right putamen, globus pallidus, ventrolateral thalamus, dorsomedial thalamus, bilateral ventroposterior lateral thalamus, cerebellum, and SMA. However, the EG movements were associated with right ventral premotor, left ventroposterior lateral thalamus, right dorsomedial thalamus, and bilateral cerebellum. The group tested output path coefficients respective of whether each movement type was associated with CBGT loops or cortico-cerebellar loops. IG involved stronger path links with the CBGT structures listed above. However, EG was associated with stronger cerebellar connectivity to ventral premotor cortex via the thalamus. The cortico-cerebellar connectivity did not involve mediation by the striatum in EG, while IG was strongly associated with putamen activity of the right (contralateral) side. So, although EG tasks appear to be less dependent on the CBGT loops, they rely strongly on cerebellar input (104). Additional work since Taniwaki et al. (104) has largely confirmed this conclusion (43, 73).

### Cerebellum

Unsurprisingly, the cortico-cerebellar system is perhaps the most cited neural pathway to be associated primarily with EG movements (41, 43, 45–47, 60, 73, 104). The importance of cerebellar feedback during EG tasks likely indicates the role of the cerebellum as a modulator of complex motor dynamics and proprioception. The cerebellum acts as a *de facto* servo system to modulate gross motor action into controlled and coordinated movement. This is also reflected in the cortical networks upstream of cerebellar activity in EG, as the frontal eye fields perform an analogous role in saccades and transitions to smooth pursuit. Additionally, these structures serve in concert with the semicircular canals and the cerebellum (particularly the flocconodular lobe) to regulate postural stability and vestibular state (105).

Imaging studies have expounded upon our understanding of cerebellar function in cued movements that had previously been largely derived from literature related to cerebellar damage (106). Functional MRI of upper extremity movement has indicated discrete lobes of the cerebellum that appear responsible for facilitating movement in response to external cues. For example, 10 healthy right-handed subjects, while fixating on a visual cross, were cued to press a button in response to hearing a sound, causing activations in lobules V and VI in the right anterior cerebellum (36). With a finger-tapping task similar to the one described above, activations were found in right lobules IV–V and right lobules VIIIA and VIIIB (37). Sauvage et al. (23) compared the neural substrates involved in execution vs. mental imagery of sequential movements (fast and slow) of the left foot in 12 volunteers. Overt movement execution and motor imagery shared a common network: premotor, parietal and cingulate cortices, striatum, and cerebellum (23). Motor imagery recruited the prefrontal cortex, and motor execution recruited the sensorimotor cortex. Slow movements recruited frontopolar and right dorsomedial prefrontal areas bilaterally in execution and motor imagery. Fast movements strongly activated the sensorimotor cerebral cortex. However, the anterior vermis, lobules VI/VII and VIII of the cerebellum were activated in fast movements, in imagery and execution (23). Fast movements are similar to ballistic movements, which have also been implicated in cerebellum imaging studies (107). These findings indicate regional functional specificity potentially exclusive to the execution of EG tasks in the upper and possibly in the lower.

Bostan et al. (108) recently showed that in the cebus monkey, the cerebellum is connected disynaptically with the subthalamic nucleus via the pontine nuclei. While previous work by the group had insinuated structural isolation of the cerebellum from the basal ganglia and STN (68), the findings of this paper clearly show a bidirectional connectivity of these regions in higher order primates. As such, modulation of the STN via cerebellar control is likely implicated in pathophysiology like PD. As described above, the STN exerts powerful effects on the basal ganglia. Given the critical involvement of the cerebellum in EG movements, it is possible that additional cerebellar input accounts for a portion of the behavioral differences of PD patients when comparing IG vs. EG task performance.

Unfortunately, few studies compare EG and IG movements in the lower extremity, which could help delineate the cerebellar components involved with each movement type. Currently, findings indicate EG movements of the lower limb, when employing comparable cues to those used to cue upper limb movements, tend to recruit both overlapping and discrete cortico-cerebellar neural structures (30, 34). Just as the cerebellum is involved in IG movements (in addition to the STC circuit), subcortical striatal structures are involved in EG foot movements. Sixteen healthy subjects performed dorsi-plantar flexion of the foot actively (responding to auditory cues at 1.25 Hz) and passively. Passive movements activated cortical regions similar (but reduced) to those activated by the active task. Activations during active and passive movement were found not only in the contralateral M1 and S1 cortices but also in the premotor cortical regions (bilateral rolandic operculum and contralateral SMA) and in subcortical regions (ipsilateral cerebellum and contralateral posterior putamen) (27). Additionally, differential activation has been noted in the cerebellum depending on whether movements are IG or EG, regardless of presence or absence of visual feedback and activation related to proprioceptive input (29). Clearly, this area of inquiry requires additional carefully planned studies to identify cerebellar circuits and auxiliary structures that can be preferentially targeted by EG interventions.

## Parkinson’s Disease and EG

Parkinson’s disease affects both IG and EG movements. However, EG movements may be less impaired in early stages of the disease as compared to IG movements (44). Interestingly, PD is associated with alterations in recruitment of cortico-cerebellar networks despite only mild overt performance differences. For example, Elsinger et al. (48) used the paced finger-tapping task (PFT) (with the right hand) and observed that PD participants had decreased accuracy and increased variability on the task compared to controls. Whether the PD participants were on or off dopamine supplementation did not affect task performance. However, decreased activation in the left sensorimotor cortex, cerebellum, and medial premotor system was noted in PD subjects compared to controls (48). In another study, PD participants (tested both on and off anti-parkinsonian medication) and age-matched controls participated in an EG sinusoidal force task with visual cues and varying speeds while gripping a squeeze bulb in their right hand. The group reported that off-medication as opposed to on-medication PD participants recruited the bilateral cerebellum and primary motor cortex as compared to on-medication PD participants and controls (40). Cerasa et al. (47) described findings from a right hand, finger-tapping paradigm of IG and EG movement with visual cues in PD patients. Both PD and healthy subjects engaged somewhat similar neural networks in both EG and IG movement, yet the PD group showed greater activity in sensory and associative cortices. For example, in the EG condition, PD subjects showed increased activation of the calcarine cortex bilaterally, potentially indicating an increased reliance on visual input for task performance. In the IG condition, the cerebello-thalamic pathway was shown to be involved to a greater degree in PD subjects, possibly denoting a compensatory modality shift to EG mapping, which is perhaps more robust to failure in PD than neurotypical IG pathways (47).

With respect to lower extremity function, recent findings have been mixed regarding cerebellar changes in PD. For example, Schwingenschuh and colleagues, using an EG ankle dorsiflexion task, found that people with PD activated lobules I–V in the ipsilateral cerebellum during EG movement, and similar cerebellar activations were found for healthy controls. After oral administration of levodopa, the PD participants showed increased activity in subcortical structures (contralateral putamen and thalamus), compared to control participants who showed no alteration of function. These findings suggest that the cortico-subcortical motor circuit in PD is sensitive to exogenous dopamine administration (25). Externally cued motor imagery has also been employed during fMRI to probe changes in cortico-cerebellar structures in gait. Cremers et al. (109) reported that people with PD compared to healthy controls had decreased activity in cerebellar vermis and SMA. Importantly, individuals with PD who exhibited greater gait disturbance were less likely to recruit cerebellar and cortical regions characterized by the healthy control group during gait visualization (109). This finding is in contrast with results of Spraker et al. who noted increased recruitment of cerebellar structures and pathways with disease progression (110). Interestingly, what might account for the varied results between studies is the relative difficulty with which studies using fMRI can quantify modulation of cortical structures by the basal ganglia. Recently, Cagnan et al. (111) demonstrated using LFPs with people with PD that phasic synchronization of basal ganglia structures is more associated with tremor and motor dysfunction in PD. Interestingly, when on dopaminergic treatment, the phase locking in beta waveforms in STN and globus pallidus abated to a more dynamic oscillation and, importantly, the individuals with PD exhibited improved motor function. Possibly, were LFPs in STN to be acquired in concert with EEG, alteration of cerebellar and cortical activity may indeed reflect oscillatory activity in the beta frequency range. In turn, combining the three techniques, while methodologically challenging, may help account for the varying reports of cortical involvement using fMRI.

At present, due to the dearth of research involving lower limb movement and imaging, drawing any conclusions about the effect of PD on neural circuits respective of EG movements in the lower extremity is challenging. Table 1 lists a summary of the relevant imaging studies in the context of IG vs. EG movement in healthy controls and individuals with PD, which were considered in the text above. Clearly, there are many questions to be answered, making this investigation important for consideration for motor and rehabilitation scientists.

## Rehabilitation Programs Focused on Rhythmic Movement

Until this point, this review has attempted to elucidate the neural mechanisms that are involved with IG and EG movements respective of the hand and foot respective of both healthy individuals and individuals with PD. We now turn to modern rehabilitative programs that may select for a movement modality or their interaction. These programs selectively engage and optimize movement using internal or external cues, or in many cases, both.

Therapy programs that include external guidance through consistent rhythmic auditory stimulation (RAS) (e.g., a metronome or music) can facilitate movements and are recommended for people with PD. RAS has been used to improve gait in those with PD via external sensory cues consisting of metronome beats. Studies have shown the positive effects of RAS on FOG and gait parameters (112). Although other sensory cues such as visual and proprioceptive cues have been examined, auditory cues appear to be most effective in improving gait in PD (113). Coupling gait to rhythmic auditory cues may rely on a neural network engaged in both perceptual and motor timing in individuals with PD (114). In fact, some individuals with PD may have an impaired perception of beat timing. Leow et al. (115) examined the impact of beat salience in effectively improving gait cadence and other parameters by comparing “high groove” (i.e., music with a strong underlying beat) to “low groove” music. Individuals with poorer perception of beat timing were helped by high groove music because of the salience of the beat. Such musical support might help facilitate gait in those with PD. This finding is highly relevant to dance- or music-based rehabilitation because poor or good beat perception affects gait performance when synchronized to music (115). Leow et al. also showed that more familiar music elicited less variable strides and faster stride velocity and better synchronization with the music (116). Salience of a beat (as mentioned above) and familiarity with music are therefore considerations for rehabilitative purposes.

Indeed, music therapies may have some utility in ameliorating some function in individuals with PD. Recently, Bella et al. trained PD participants on musically cued gait therapy, consisting of synchronizing movement to familiar folk music without lyrics (a bell cued participants’ movement). Findings included not only increased gait speed and stride length but also strong gains in motor synchronization (tapping) and perceptual awareness on just noticeably different tasks (117). Findings from stroke literature and the application of music-supported therapy (MST) might also shed light on possible beneficial effects of rhythmic movement with auditory support for people with PD. MST uses musical instrument playing to treat paresis of the upper limb and adheres to four principles: massive repetition, audio-motor coupling, shaping, and emotion–motivation effects. After 4 weeks of MST in combination with usual care, chronic stroke participants assigned to MST showed improvements on the Wolf motor function test in comparison to a control group (118). Additionally, a case study revealed audio-motor coupling when a patient was exposed to a passive listening task with unfamiliar and trained melodies. Before MST, only the auditory cortices were activated; after MST, motor regions were also activated (119). Given that participants actually play an instrument, MST is an intriguing form of musical therapy that can exploit benefits of both EG and IG strategies, and their accompanying neural circuitry.

A meta-analysis recently demonstrated that music-based therapy, including dance, positively affects PD gait and gait-related activities (120). Recently, dance has indeed gained attention as a music-based therapy that may be able to effectively address impairments related to PD. An understudied aspect of dance interventions is the interplay of external vs. internal guidance across multiple sensory modalities. Proprioceptive and kinesthetic inputs based on tactile cues are crucial for motor adaptation and dance performance. Visual cues no doubt play a role in postural control, navigation, and emotional understanding, as well as having a curious positive effect on FOG. However, auditory cues (e.g., percussion or other musical rhythms) play a strong role in guidance of movement and can be EG (e.g., bass percussion) or IG [e.g., fermata pause (notes held longer than music’s tempo)] or even delivered as disruptive asynchrony (e.g., syncopation). We believe rehabilitation regimens like dance and other rhythmic training likely provide a synergistic multisensory adjuvant to motor skills training in both aging and disease models. However, in consideration of the discussion of the neural pathways associated with IG and EG motor training strategies, answering the question why dance may be effective is helpful. At this point, it is unclear to what extent external musical auditory or visual (and tactile, in the case of any sort of partnered/contact dance) cues play to elicit therapeutic effects vs. the improvements gained by increased attention and cognitive engagement used to plan and enact movement. Studies are needed to answer these questions when considering the research that has accumulated supporting beneficial effects of rehabilitative methods involving rhythmic training.

### Dance Therapy

In the last 10 years, a series of studies have investigated the effects of adapted Argentine tango dance (adapted tango) for individuals with PD. Participants experienced significant gains in mobility, balance, and QOL (121–124). These improvements were maintained 1 month later, (123) and up to 3 months later (125, 126). After participating in 1 year of tango classes offered in the community, participants with PD also demonstrated decreased disease severity (127). Recently, a study demonstrated a 12-week adapted tango program, which was disseminated to several novice instructors and offered in the community, improved spatial cognition, as well as disease severity in participants with mild-moderate PD (126). Other forms of dance have been investigated for efficacy for those with PD. A study that investigated the feasibility of Irish set dancing, in comparison to standard physiotherapy, found the dancing safe and feasible. Furthermore, participants tended to improve more in gait, balance, and FOG after dancing, than after the standard care (128). Dance may have an immediate effect on mobility in those with PD as improvements have been found in as little as 2 weeks of tango (129) and contact improvisation training (130). The very popular “Dance for PD” method has been investigated for its efficacy, and it was found to improve the motor subscale of the UPDRS after 16 sessions (20 h of treatment) in an uncontrolled study (131).

Because several studies have been conducted on the efficacy of tango, it will be examined and considered for its qualities, to serve as an exemplar with qualities that can relatively easily be identified within the IG/EG dichotomy. Argentine tango has steps, patterns, music, and importantly, partnering that may address specific impairments associated with PD. Partner dancing is a sophisticated, yet accessible system of tactile communication that conveys motor intentions and goals between a “leader” (planner of movement) and “follower” (externally cued mover). An “embrace” or “frame” between the leader and follower is the position maintained by the arms throughout all steps in adapted tango. In adapted tango classes (121, 123, 125), participants consistently both led and followed all dance steps with healthy partners, and therefore alternated between two motor training approaches (a) leading, consisting of internally guiding movement plans and (b) following, consisting of responding to external guidance. Thus, qualities of effective rehabilitative programs are found in both leading and following within the context of adapted tango. While in the role of leader, participants practice self-directed, internally generated movements; while, in the role of follower, participants practice responding to external cues from the partner. There are key differences between leading and following that may address specific needs and result in distinct training gains in mobility, because as we have outlined above the neural circuitry that drives leading and following movement likely differs.

Individuals who perform the leading role in dance are thought to adopt a world-centric reference frame. To lead a dance successfully, these individuals need to multi-task by focusing on environment, follower, music, and both current and future motor plans. Leading, which should be using IG cognitive and motor skill, is thought to involve employing a “movement strategy” that demands increased focus on movement plans and mentally rehearsing and/or preparing for movement. Leaders in partnered dances must determine precise spatiotemporal movement parameters of a dance sequence, e.g., amplitude, direction, timing, and rotation. As such, leading may pose a challenge for individuals with PD, given that many have impaired executive control, specifically in cognitive processes involved in planning and executing complex, goal-directed behavior (11, 132). Importantly, the individual who follows in adapted Argentine tango is not required to plan precise spatiotemporal parameters of movement (e.g., direction, length of step, timing, and amount of rotation). From moment to moment, the follower receives movement guidance regarding the afore-mentioned parameters from the leader via tactile cues. Because followers are not devoting attentional resources to planning movement, potentially they can attend more to their postural control, which becomes more and more necessary as a person ages or contends with a neurodegenerative movement disorder.

Although the adapted tango dichotomy of leading and following roles provides a convenient analog to the “ideal” EG and IG motor training vehicle, it must be admitted that underlying strongly rhythmic movements that characterize dance forms continuously employ both EG and IG strategies. Whether these rhythms are internally created or manifested from external guidance (from music and tactile cues of a leader), dance movements obey their inherent timing. As such, rhythmic cues may be very responsible for any gains seen in PD rehabilitation as a result of dance participation. However, in practice, it can be challenging to both train a person with PD (or even a “healthy” individual) in complex shapes of a dance form and also teach them the sophisticated rhythms that make up most dances. As such, the consideration of shape vs. timing must be acknowledged.

The “shape” of a movement sequence is the simple biomechanics of the steps, irrespective of speed or timing. Rhythm usually refers to a repetitive pulse that is repeated in cycles through a musical or movement form. Even IG movement, which does not appear to “obey” a particular repeated rhythm has an intrinsic timing, and occupies a temporal space [see Ref. (75)]. In a dance class for people with motor challenges, there are a number of stages through which a dancer may go in order to achieve dance mastery, which includes a mastery of coordinating movement to music. This movement entrainment may or may not be the same as reports from Wu et al. (96) investigating finger movement training to automaticity, as coordinated rhythmic movements involve a much more complex interplay of IG and EG timing. During rhythmic training, in the first/novice stage, the individual begins to understand a dance pattern, and puts their body through novel motions that will occupy some sort of temporal space, but may not align precisely with a dictated rhythm/timing given by an instructor. With practice the dancer enters a second stage, in which he/she has the motor control to coordinate his bodily timing to the musical timing. As the steps become more complex, there is vacillation between the first and second stages. But whether or not the dancer (a) benefits from precisely moving to the beat of the music, or the dance rhythm (b) from listening to the music, and/or – thinking – they are moving precisely to the beat and obeying a dance rhythm, or (c) benefits mostly from concentrating on the shape of the movement, while creating their own internal rhythmic timing, is unclear.

The role of the instructor is extremely important with respect to properly implementing adapted rhythmic training programs, as the trained instructor becomes the model by which the individual gages performance. During initial instruction, the student models performance externally by visual approximation of his or her own movement to that idealized by the instructor. As the student progresses and achieves additional kinesthetic feedback and motor flow during the rhythmic movement, motor adaptation translates from externally derived imitation to IG motor flow. However, despite this transition, the role of the instructor to provide the movement template is central to successful training. In these regards, the student’s interpersonal relationship with their instructor has great import on their training trajectory; however, this relationship necessarily introduces variability due to social interaction between student and teacher. Interestingly, recent work has approached the interpersonal dynamics involving adapted tango. A research group at Emory University and Georgia Tech has been investigating the ability of robots to act as leaders and followers in a simple tango step pattern. The robots are able to maintain a stable distance from their human partner, characteristics of a human counterpart. While experiments using these robots are ongoing, a recent report demonstrated that expert dancers have indicated reasonable ecological validity of the leading and following performance of the robots (133). This line of work offers unique insights as to the effects of interpersonal dynamics on rehabilitative outcome using dance therapy. Furthermore, these investigations could serve as an interesting platform upon which to test ideas about IG and EG movement schemas.

## Rhythmic Movement in Rehabilitation of Internally and Externally Guided Movement

At this time, few studies offer evidence of neural changes as a result of focused training in people with PD or healthy controls. A study utilizing PET showed improved vocal intensity after training in the Lee Silverman voice training (LSVT) LOUD program for speech improvement. These motor improvements were correlated with modification in motor, auditory, and prefrontal areas, but there was no effect on the basal ganglia (134). However, in healthy participants lying supine, increased activity in the putamen was noted using PET when tango movements were performed with a single limb to a metered beat (135). In a related finding, after a week of tango lessons, healthy adults exhibited increased activity of supplementary motor (SMA) and premotor cortices during imagined tango-style walking. In participants who had completed a week of locomotor attention training involving physical and mental practice, activation was examined during an overt foot motor task consisting of ankle dorsiflexion. Posttraining the foot task showed reorganization of sensorimotor areas, in keeping with other studies on lower limb motor learning, suggesting that functional connectivity of the sensorimotor network may be modulated by focusing attention on the movements involved in ambulation (136, 137). Dobkin et al. (24) assessed how ankle dorsiflexion could be utilized as an fMRI paradigm to measure the efficacy of a rehabilitative strategy – body weight-supported treadmill training – for hemiparetic subjects. During voluntary ankle movement, the subjects completed two sets of five isolated movements of 10°. The study observed reorganization in the brain with training; specifically, decreased activity in the ipsilateral primary sensorimotor cortex (S1M1) as therapy gains increased and by 2–6 weeks of training, three of the four subjects saw increased activity in the contralateral S1M1 (24). More studies are clearly needed to examine neural changes in combination with observed clinical motor and cognitive changes, particularly with respect to particular motor training strategies.

## Limitations

As stated earlier, there is a paucity of research investigating the neural correlates of lower limb movements in aging and disease. As such, much of the research concerned with initiation of movement from an IG vs. EG perspective is derived from work in the upper extremity. While we have noted overlap of cortical, subcortical, and cerebellar structures when comparing IG against EG movements of the hand and foot, considerable variation has also been reported particularly regarding laterality of movement. While this variation might, in part, limit interpretation in the current review, its presence provides a direct opportunity for inquiry in both basic science and applied rehabilitation investigations. In addition, a challenge that Dobkin et al. have described in imaging-related literature in rehabilitation is the problem of describing neural activity using BOLD imaging respective of time of data acquisition – both post-onset of pathology and progression through the rehabilitation regimen (24). The group has provided evidence that neural activity during early training tends to be larger in volume. However, with increasing exposure, despite similar task performance, neural activity appears to be consolidated to smaller cortical volumes. This presents a challenge in the review of rehabilitation literature as simple static statistical threshold comparisons of activity volumes across studies conflate the neurological variability of the substrates under consideration due to both age and the rehabilitation regimen. Again, due to the low number of rehabilitation studies comparing IG vs. EG in the imaging literature, we lack the statistical tools to describe this variability properly in the current review. Perhaps most importantly, in the current review, we have not discussed the critical aspect of dosing of rehabilitative regimens engaging in EG and IG movement strategies. The identification of an optimized amount of treatment has been overlooked in rehabilitation research. This may have been of necessity given the field has been engaged in identifying specific regimens for efficacy. However, given recent success in identifying programs, it is appropriate to begin to ask the question, “how much?” and to probe for differences between IG and EG motor training with respect to dose. Furthermore, this review has only very briefly outlined the investigation into music-based and dance-based therapies within the PD population and has likely not thoroughly covered the rhythmic and cognitively driven (IG) aspects of other forms of exercise (e.g., spinning, Tai Chi, walking, swimming, and boxing), which deserve attention.

## Conclusion

Underlying mechanistic commonalities may exist among therapies that effectively target symptoms of individuals with PD (138). As a jumping off point, one can consider the effects of levodopa and deep brain stimulation, the current standard of care treatment for today’s population with PD. As with the case of deep brain stimulation, suppression of abnormal downstream network activity produced by the malfunctioning basal ganglia may result from stimulating the subthalamic nucleus (139). Rehabilitation may also create changes downstream of basal ganglia structures. If the mechanism of improvement resulting from IG motor training is similar, there may be a reduction in abnormal neural activity along the STC circuit, which likely mediates IG motor tasks and includes the putamen, ventral anterior thalamus, rostral SMA, and primary motor cortex. An alternative possibility could be increased activity in the basal ganglia, which have been demonstrated to be hypoactive in drug-naïve individuals in early stages of PD (140). Moreover, when examining twins discordant for PD performing right hand, finger sequencing tasks for task specific influences on the STC and CTC pathways before and after levodopa administration, it was noted that levodopa corrected hypoactivation in the contralateral STC, but over-corrected activation in the ipsilateral STC and bilateral CTC pathways; therefore, standard PD pharmacology affects compensatory changes (51) and effective IG or EG motor training as well. Nevertheless, currently, insufficient evidence exists to determine the mechanisms by which IG and EG motor training are efficacious, and future work is necessary to do so. Furthermore, to understand the mechanisms underlying impairments and training effects in whole-body balance and mobility tasks, lower limb neural activity must be investigated first within the context of IG and EG tasks in individuals with and without PD. Knowledge about neural changes that may occur after repeated and targeted training with IG or EG tasks will allow us to develop better rehabilitation training strategies for those with PD and supplement pharmacological and surgical developments.

## Author Contributions

MH and KM drafted the manuscript, performed literature review, and edited the final manuscript for submission. HL and JB contributed literature search and review and tabular organization. BC performed final review and critical appraisal of the manuscript.

## Conflict of Interest Statement

The authors declare that the research was conducted in the absence of any commercial or financial relationships that could be construed as a potential conflict of interest.
